# Effect of Spatial Orientation of Fibers on Fracture Resistance of Large Mesio-Occluso-Distal Cavities in Maxillary Premolars: An In Vitro Study

**DOI:** 10.7759/cureus.94733

**Published:** 2025-10-16

**Authors:** Garima Vatsa, Chi Koy Wang, Somnath Pal, Abhinav K Singh, Abhishek Kumar

**Affiliations:** 1 Department of Conservative Dentistry and Endodontics, Buddha Institute of Dental Sciences and Hospital, Patna, IND

**Keywords:** composite restoration, fiber orientation, fracture resistance, mod cavity, polyethylene fibers

## Abstract

Aim: The aim of this study is to determine the outcome of fiber insertion on the fracture resistance of composite restoration in wide mesio-occluso-distal (MOD) cavities in maxillary premolars.

Materials and methods: After selection of 60 extracted maxillary premolars, large class two MOD cavities were prepared, and samples were divided into five groups (n=12) for insertion of polyethylene fibers. In group A, fibers were placed horizontally in the mesiodistal direction, while in group B, they were placed buccolingually. In Group C, they were placed in a U-shaped design, in Group D, it was placed in a circular orientation, and in Group E, there were no fibers. After the completion of the composite restoration and incubation process, the samples were tested under a universal testing machine at a crosshead speed of 1mm/min to measure the fracture resistance of all the samples, and the values were obtained in Newton (N).

Results: The fracture resistance was highest in Group A (910.58 N), followed by Group C (898 N), Group B (894.33 N), and Group D (753.08 N). Group E (without fiber) showed the least fracture resistance value (549.25 N).

Conclusion: Fiber reinforcement significantly improves the fracture resistance in maxillary premolars, with Group A having superior results as compared to others in the present study.

## Introduction

The objective of restoration is to bring the tooth to its normal form, function, and esthetics. Restoring extensively carious teeth where both the marginal ridges are involved is a demanding situation in the field of operative dentistry. The fracture resistance and tooth stiffness are governed by various factors. A direct relationship exists between the amount of remaining tooth structure and the ability to resist occlusal forces. The major change in tooth biomechanics is due to loss of hard tissues following decay, fracture, and cavity preparation. The largest reduction in tooth stiffness is especially due to the loss of marginal ridges. The literature reports approximately 20% to 63% reduction in tool stiffness following mesio-occluso-distal (MOD) cavity preparation [1,2].

After the development of the adhesive bonding technique, restorations can give support to the remaining tooth structure rather than gain support from the tooth. Direct composite restorations are popular due to their esthetic property and bonding ability to tooth structure [3]. The significant increase in the use of low-shrinkage composites for posterior restoration can be attributed to their minimal microleakage and improved mechanical properties [4].

In recent years, the use of fiber-reinforced composite resin restorations in dentistry has become popular. There is a wide variety of commercially available fibers in the market; among them is the “Leno-Woven polyethylene ribbon” fiber. Using this fiber along with a composite strengthens the structurally compromised teeth [5]. The Leno-woven ultra-high-molecular-weight polyethylene continuous fiber ribbon system enhances the toughness of the composite, thereby making it more tolerant to damage [6,7]. The continuous fibers have yarns arranged in multiple directions and a mesh-like pattern with nodal intersections, which serve as a path for the redistribution of masticatory forces over a large bed of composite [8].

Various in vitro studies have demonstrated improved fracture resistance of heavily restored and endodontically treated teeth that incorporate long polyethylene fibers, which act as a stress absorber, effectively distribute the forces, and prevent crack propagation [9,10].

Meiers et al. have shown that composite restorations with woven polyethylene fibers have superior tensile and compressive strength as compared to non-fiber restorations [8]. Another investigation by Garoushi et al. revealed that fibers, when placed in the deepest part of composite restoration, have a positive effect on their fracture strength [11].

This study has been done to evaluate the outcome of horizontal (mesiodistal), horizontal (buccolingual), U-shaped, and circular orientation of fibers on fracture resistance of large MOD cavities.

The null hypothesis tested was that the different spatial orientations of fibers in composite restoration do not affect their fracture resistance.

## Materials and methods

Study design

**Figure 1:**
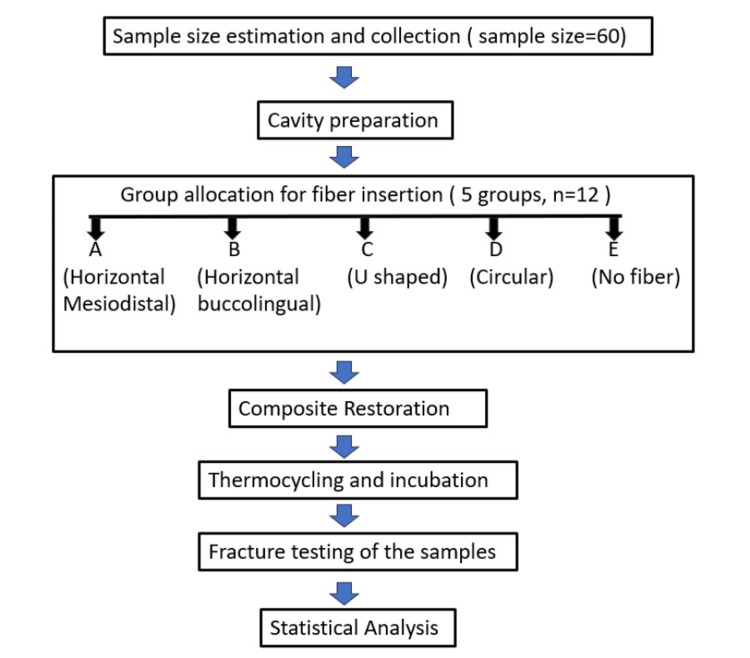
Study flow diagram

An in vitro study was designed to determine the outcome of fiber insertion on fracture resistance of composite restoration in wide MOD cavities in maxillary premolars. The study protocol was approved by the Institutional Ethical Committee, Buddha Institute of Dental Sciences & Hospital, Patna, Bihar, India (Ref. No. 135/BIDSH). The flow diagram provides an overview of the study (Figure 1).

Source of samples and sample size estimation

Maxillary premolar teeth extracted from patients undergoing orthodontic treatment were used in this study. Sample size estimation was done using G*Power statistical software (version 3.1.9.7, Heinrich-Heine-Universität Düsseldorf, Germany). The effect size (f=0.54) was derived from the pooled mean (917.47N) and pooled standard deviation (158.59N) of fracture resistance values reported in a previous study [12] on fiber-reinforced composite restoration. A minimum sample size of 50 specimens was estimated for five groups with an alpha error of 0.05 and 80% power. The final sample size for the five groups was increased to 60 (12 per group). Sixty extracted maxillary premolars collected from the Department of Oral and Maxillofacial Surgery were cleaned, and any deposited calculus was removed using an ultrasonic scaler (Woodpecker, China). Teeth were stored in distilled water until preparation. Teeth with cracks and fractures, pulpal exposure, and restored and endodontically treated teeth were excluded from the study.

Cavity preparation

**Figure 2:**
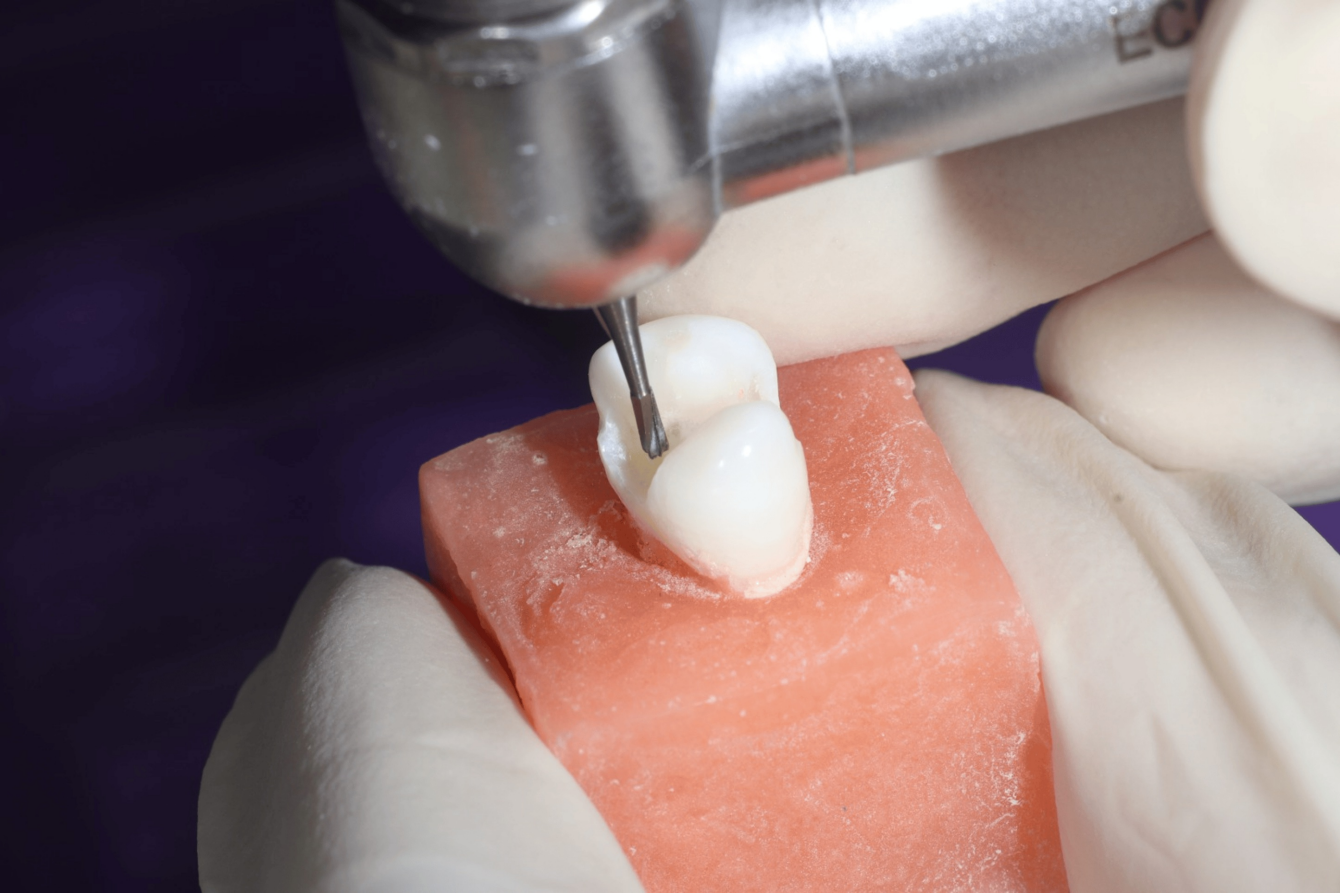
MOD cavity preparation MOD: Mesio-occluso-distal

All the samples were mounted in cold-cure acrylic resin (DPI, RR, India) blocks. To simulate the periodontal ligament, the root was covered with a silicone impression material (Avue, Korea) up to the cemento-enamel junction. Class II MOD cavities were prepared on all the samples by a single operator with a depth of 3mm, and the width was kept one-third of the intercuspal distance with the help of an airotor handpiece (Alegra, W&H, Austria) and a 245 carbide bur (Prima, India) (Figure 2). The depth of the cavity was checked using a Williams probe (API, Germany). After the cavity preparation, the matrix band was adapted using a Tofflemire retainer (API, Germany).

Fiber placement and composite restoration

**Figure 3:**
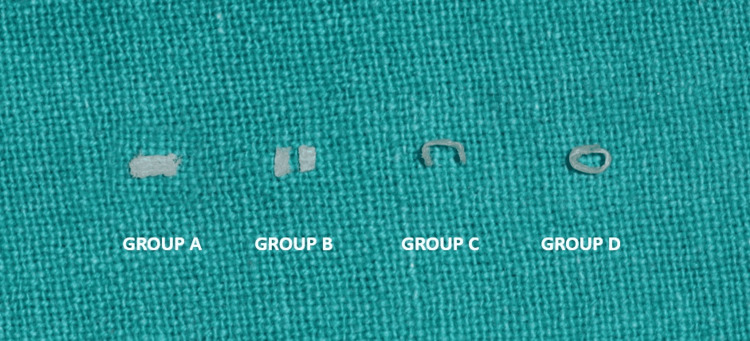
Different orientations of fibers

Teeth were etched with 37% phosphoric acid gel (Eco-etch Ivoclar, Liechtenstein) for 15 seconds and washed with distilled water and air dried. Bonding agent (Te-Econom Bond, Ivoclar Vivadent, Liechtenstein) was applied on the prepared tooth surface with the help of an applicator tip and cured with a light curing unit (Bluephase NG4, Ivoclar) for 10 seconds. A thin layer of flowable composite (3M Filtek, USA) was placed on the pulpal floor but not light-cured. The samples were divided into five groups for the placement of fibers in different orientations (Figure 3). Ribbon fibers (Ribbond, Seattle, USA) were wet with unfilled resin (Ribbond Wetting Resin,USA) and thereafter placed according to the orientation in different groups as follows: Group A: the ribbond fiber was placed on the pulpal floor in horizontal direction mesiodistally; Group B: the ribbond fiber was cut to measurement in accordance with the width of the cavity buccolingually and placed horizontally in the buccolingual direction; Group C: the ribbond fiber was placed on the pulpal floor and also adapted over both the axial wall in a U-shaped orientation; Group D: the ribbond fiber was adapted around the cavity walls in a circular orientation; Group E: cavity restored only with composite resin without any fiber placement (positive control).

After the arrangement of fibers in different orientations over the flowable composite, light curing was done for 20 seconds. Composite resin (3M Filtek, USA) was condensed into the cavity with the help of composite filling instruments (LM Arte) in increments and light-cured. After the completion of the restoration, the Tofflemire retainer and matrix band were removed. Finishing and polishing of the restorations were done with composite finishing (Shofu, Super Snap, Japan) and polishing kit (Eve Diacomp, Germany) using a contrangled handpiece (NSK, Japan). 

Fracture testing

**Figure 4:**
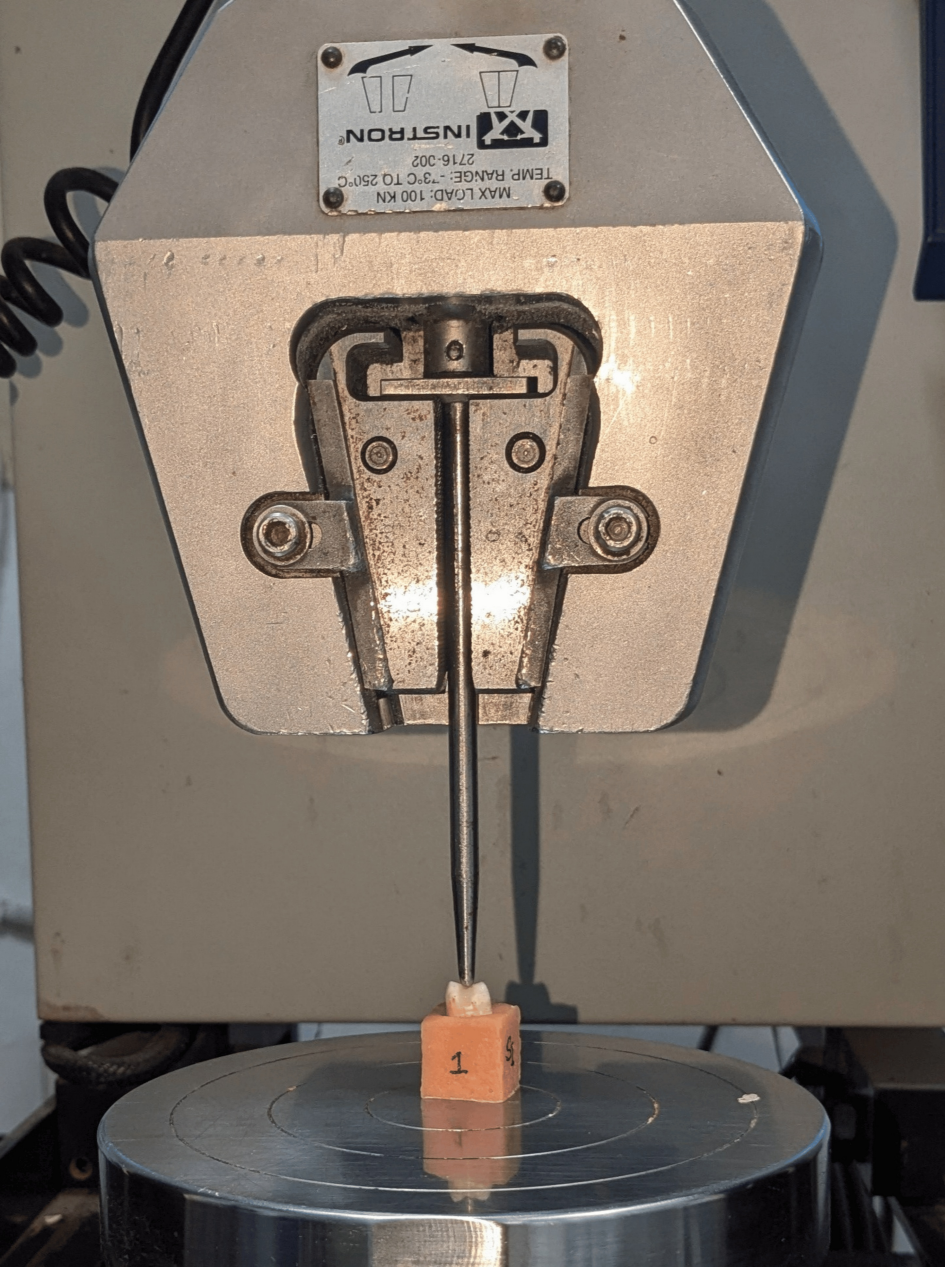
Testing of the sample for fracture resistance in the universal testing machine

After the composite restoration, all teeth were thermocycled for 500 cycles at 5-55 degrees Celsius with a 30 seconds dwell time. Before testing, the samples were stored in distilled water at 37 degree Celsius for 24 hours. The fracture resistance of each specimen was checked in the Universal Testing Machine (Figure 4) with an indenter diameter of 5mm at a speed of 1mm/minute, and the values were recorded in Newton (N).

Standardization, calibration and reliability testing

To ensure accuracy and consistency, all cavity preparation and restorations were done by a single operator to eliminate inter-operator variability. The dimensions of the cavities were verified using William's probe (API, Germany). The bur was replaced after every five-cavity preparation to ensure cutting efficiency and minimize variation in cavity surface texture. A restorative dentist with over 10 years of clinical and academic experience participated in the study as an examiner and was familiarized with the study design. To calibrate the examiner, a session was conducted on a separate set of pilot samples (n=10), which were prepared and evaluated similarly to the study samples. To prevent bias, the examiner was blinded to the group allocation. For intra-examiner reliability assessment, 15 randomly selected samples were re-examined at an interval of one week. The scores obtained from these two evaluations were analyzed using Cohen's Kappa (k) statistic to determine the consistency. A k-value of 0.78 represents a good agreement and substantial consistency.

Statistical analysis

Descriptive and inferential statistics were analyzed using IBM SPSS Statistics for Windows, Version 26 (Released 2019; IBM Corp., Armonk, New York, United States). The Shapiro-Wilk test was used to test the normality of the dataset, which revealed that the data were not normally distributed. Hence, the Kruskal-Wallis test was done to determine the statistical significance, followed by Dunn's post hoc pairwise comparison. A p-value of <0.05 was considered a statistically significant difference.

## Results

The highest mean fracture resistance was found in Group A, followed by Groups C, B, D, and E. Table 1 presents descriptive statistics of fiber placement in different orientations.

**Table 1:** Descriptive statistics and variability n: number of samples in each group; N: Newton

Descriptive Statistics								
	n	Range	Minimum	Maximum	Mean (N)		Std. Deviation	Variance
	Statistic	Statistic	Statistic	Statistic	Statistic	Std. Error	Statistic	Statistic
Group A - Fiber placed horizontally in the mesiodistal direction on the pulpal floor.	12	271.00	739.00	1010.00	910.58	27.68	95.90	9197.35
Group B - Fiber placed horizontally in the buccolingual direction on the pulpal floor.	12	200.00	798.00	998.00	894.33	12.40	42.97	1847.15
Group C - U-shaped orientation of fiber on the pulpal floor and axial wall.	12	375.00	680.00	1055.00	898.00	32.24	111.69	12475.27
Group D - Circular orientation of fiber	12	250.00	645.00	895.00	753.08	15.57	53.94	2910.44
Group E- Positive control cavity restored only with composite resin without any fiber placement	12	125.00	476.00	601.00	549.25	10.89	37.72	1423.47

Group A (fiber placed horizontally in the mesiodistal direction) exhibited the highest mean value (910.58N), indicating superior reinforcement among the tested groups. Group B (fiber placed horizontally in the buccolingual direction) and Group C (U-shaped orientation) had slightly lower mean values (894.33N and 898N, respectively), suggesting comparable but slightly reduced reinforcement effectiveness compared to Group A. Group D (Circular orientation) showed a significantly lower mean value (753.08N), indicating lesser reinforcement than the horizontal and U-shaped orientations. Group E (Positive control without fiber placement) recorded the lowest mean value (549.25N).

The normality of data distribution was tested using the Shapiro-Wilk test. The significance (Sig.) values from this test indicate whether the data follows a normal distribution (p > 0.05 suggests normality, while p < 0.05 suggests deviation from normality).

The Kruskal-Wallis test, the non-parametric equivalent of one-way ANOVA, was performed to determine whether statistically significant differences existed in the median fracture resistance among the five groups. The test yielded a p-value of 0.0001. Since p<0.05, it led to the rejection of the null hypothesis and confirmed a significant effect of fiber orientation on fracture resistance.

The Dunn's post hoc pairwise comparisons reveal statistically significant differences among various fiber placement groups, highlighting the impact of fiber orientation on composite restoration performance (Table 2).

**Table 2:** Pairwise comparisons of fiber placement groups * denotes being statistically significant

Comparison (Sample 1 vs. Sample 2)	Test Statistic	Std. Error	Std. Statistic	p-value (Sig.)
E (Control) vs. D (Circular)	15.000	7.126	2.105	0.035*
E (Control) vs. B (Buccolingual)	30.458	7.126	4.274	0.0001*
E (Control) vs. C (U-Shaped)	36.708	7.126	5.151	0.0001*
E (Control) vs. A (Mesiodistal)	37.833	7.126	5.309	0.0001*
D (Circular) vs. B (Buccolingual)	15.458	7.126	2.169	0.030*
D (Circular) vs. C (U-Shaped)	21.708	7.126	3.046	0.002*
D (Circular) vs. A (Mesiodistal)	22.833	7.126	3.204	0.001*
B (Buccolingual) vs. C (U-Shaped)	-6.250	7.126	-0.877	0.380
B (Buccolingual) vs. A (Mesiodistal)	7.375	7.126	1.035	0.301
C (U-Shaped) vs. A (Mesiodistal)	1.125	7.126	0.158	0.875

The control group (E), which had no fiber placement, showed significantly lower values compared to all experimental groups (p < 0.05), confirming that fiber reinforcement enhances strength. Among these, Groups A (Mesiodistal) and C (U-Shaped) exhibited the highest statistical difference from the control (p = 0.0001), indicating their superior reinforcement effect.

Comparisons between Group D (Circular) and other fiber placement groups also showed significant differences, with Groups A and C performing significantly better than Group D (p < 0.05). This suggests that circular fiber placement provides relatively less reinforcement compared to mesiodistal and U-shaped configurations.

However, the differences between Groups B (Buccolingual) and C (U-Shaped) or A (Mesiodistal) were not statistically significant (p = 0.380 and p = 0.301, respectively). Similarly, Group C (U-Shaped) vs. A (Mesiodistal) showed no significant difference (p = 0.875), indicating that these orientations provide comparable performance.

Overall, these findings suggest that fiber placement significantly improves fracture strength of composite restoration.

## Discussion

There has been a paradigm shift in the field of restorative dentistry from basic mechanical retention to adhesive techniques [13]. Biomimetic dentistry aims to restore a tooth’s original biomechanical integrity with materials that closely resemble its physical and mechanical properties [14,15].

The dentin-enamel junction (DEJ) unites two very dissimilar materials having dissimilar elastic properties: enamel and dentin [16]. The DEJ, or dentinoenamel complex (DEC), brings these two dissimilar materials into a co-ordial relationship which enables harmonious transfer of stress between enamel and dentin. DEC acts as a barrier to cracks formed in enamel, which otherwise can cause catastrophic tooth fracture [17].

Premolars are more likely to be subjected to lateral forces with a more detrimental nature. Bearing in mind their position in the aesthetic zone, aesthetic requirements should be fully achieved when restoring upper premolars. In addition, the width of tooth preparation and loss of marginal ridge influences cusp fracture of these teeth in such a way that the MOD cavity is considered the worst case in terms of fracture resistance [18]. Therefore, in this study, the preparation of the MOD cavity was considered for the simulation of the worst clinical situation. In this study, deep MOD cavities were restored with direct composite restoration.

As modern composite resin materials are rigid, they do not lack strength, but they lack toughness [17]. A material with high fracture toughness can better resist crack initiation and propagation, thus would be ideal to reinforce the dentin core [19]. Polyethylene fibers possess a dense concentration of fixed nodal intersections that assist in maintaining the integrity of fibers. This enables the stresses in the bulk of the material to be transferred more effectively because of the well-defined load paths from one area to another [20].

Fennis et al. compared the fracture resistance of maxillary premolars but did not find any significant difference between composite restorations reinforced with unilateral single buccopalatal fiber and restorations with two fibers in the buccopalatal and mesiodistal direction [21].

Contrary to this, Belli et al. evaluated the effect of fibers in the fracture resistance of root canal-treated teeth and showed that the use of polyethylene fibers at the bottom or upper part of MOD cavities had a significant effect in increasing the fracture strength of specimens [22].

The result of the present study was statistically significant, and the highest fracture resistance was exhibited by Ribbond fiber placed horizontally in the mesiodistal direction (Group A, 910.58 N), followed by Group C (898 N), Group B (884.33 N), Group D (753.08 N), and Group E (549.25 N)

The results showed the highest fracture resistance for Group A (910 N), in which fibers were placed horizontally in the mesiodistal direction. This can be attributed to the coverage of a large surface area and an inherent dense network of locked nodal intersections of polyethylene fibers, which serves as a potential crack stopper.

Group A shows superior results as compared to Group B (884.33N), in which fibers were placed horizontally in bucco-lingual direction. This is in accordance with Agrawal et al., who also reported that polyethylene fiber provided better fracture strength in the mesiodistal direction as compared to the buccolingual direction [12]. Moezizadeh et al. in their study also found that fiber positioning in mesiodistal orientation showed higher fracture resistance [23].

Group C, in which fibers were placed in ‘U’ shaped orientation on the pulpal floor and axial wall, shows a mean fracture resistance of 898 N. This value is not much less than compared of group A. The reason behind this could be that although both the axial walls are connected but the fibers are not stretched and not under tension. The fibers that are adapted to the axial wall are parallel to the compressive load that has been applied [24].

Previous studies by Dyer et al. [5] and Ellakwa et al. [24] have pointed out that the placement of fibers at the tensile side improves flexure properties. Fibers increase the strength of restoration if the compressive forces are perpendicular to the long axis of fibers, but when parallel, they lead to matrix failure and no enhancement in strength [23].

Another fiber reinforcement technique in Group D is the circular orientation, which has significantly increased the fracture resistance (753.08 N) of the tooth as compared to the non-fiber group (Group E, 549.25 N). This is in accordance with the study conducted by Nvimipour et al., who reported that fiber, when placed circumferentially, increased the fracture strength of resin composite restoration [25]. Sary et al., too, in their study, found that placement of fibers in circular orientation was beneficial in improving the fracture strength of teeth [26]. Contrary to the result of these studies, Akman et al. [20] reported that the long fiber did not have a significant effect on fracture strength when applied with the circumferential technique compared with the only resin composite group.

The circular placement of fibers in Group D increased the fracture resistance, but it was significantly less than other fiber groups. This can be attributed to the fact that when fibers were adapted circumferentially, the compressive load is not perpendicular to the fiber matrix, instead, it is parallel, and in such a case, the fibers are not under stress.

The clinical implications of this research underscore the significance of fiber reinforcement in composite restoration, with particular emphasis on the influence of different fiber orientations in restoring carious teeth with substantial structural loss. The findings highlight the advantage of specific fiber orientation, which supports the matrix, enhancing stress distribution, minimizing crack propagation, and ultimately improving the overall strength of the restoration.

This research is subject to certain limitations. Notable one is the use of static load to determine the fracture resistance. The study was designed as an in vitro investigation; thus, the exact replication of a clinical condition is not possible. Given the mentioned shortcomings, the proposed technique requires a larger number of trials in the future and testing with cyclic dynamic loading.

## Conclusions

Within the limitations of this in vitro study, it can be concluded that MOD cavity preparation results in substantial loss of tooth structure, while the incorporation of polyethylene fibers in class II composite restoration significantly increases the fracture resistance. Furthermore, the present study demonstrated that horizontal placement of fiber in the mesiodistal direction on the pulpal floor of wide MOD cavities gave the highest fracture resistance in maxillary premolar.
